# Non-SMC condensin I complex subunit H mediates mature chromosome condensation and DNA damage in pancreatic cancer cells

**DOI:** 10.1038/s41598-019-54478-3

**Published:** 2019-11-29

**Authors:** Jae Hyeong Kim, Yuna Youn, Kyung-Tae Kim, Gyubeom Jang, Jin-Hyeok Hwang

**Affiliations:** 1 Department of Internal Medicine, Seoul National University Bundang Hospital, Seongnam-si, Gyeonggi-do 13620 Republic of Korea; 2 Research Institute, National Cancer Center, Goyang-si, Gyeonggi-do 10408 Republic of Korea; 3 Department of Internal Medicine, Seoul National University College of Medicine, Seoul, 03080 Republic of Korea

**Keywords:** Targeted therapies, Apoptosis, Chromosome segregation

## Abstract

Non-SMC condensin I complex subunit H (*NCAPH*) is a vital gene associated with chromosome stability and is required for proper chromosome condensation and segregation. However, the mechanisms through which NCAPH affects pancreatic cancer (PC) and its molecular function remain unclear. In this study, we examined the role of NCAPH in PC cells. Our results showed that NCAPH was overexpressed in clinical PC specimens (GEPIA) and cell lines. In addition, in NCAPH-knockdown cells, colony formation and proliferation were inhibited, and the cell cycle was arrested at the S and G_2_/M phases owing to failure of mature chromosome condensation (MCC) in poorly condensed chromosomes. Increased cell death in NCAPH-knockdown cells was found to help initiate apoptosis through the activation of caspase-3 and PARP cleavage. Furthermore, NCAPH-knockdown cells showed an increase in chromosomal aberrations and DNA damage via activation of the DNA damage response (Chk1/Chk2) signaling pathways. These data demonstrated that NCAPH played an important role in cell cycle progression and DNA damage by maintaining chromosomal stability through progression of MCC from poorly condensed chromosomes. Ultimately, NCAPH knockdown induced apoptotic cell death, which was partially mediated by caspase-dependent pathways. These findings highlight the potential role of NCAPH as a therapeutic target for PC.

## Introduction

More than 90% of pancreatic cancer (PC) cases are classified as pancreatic ductal adenocarcinoma (PDA), one of the most lethal human malignant tumors with a poor prognosis^1^. The 5-year relative survival rate for patients with PC was less than 8% during the most recently reported study period (2007–2013)^2^. Unfortunately, compared with that for other malignancies, the survival rate of patients with PC has not improved in recent decades^3^. Typically, patients are diagnosed with PC at a late stage of the disease, and the spread of highly metastatic PC cells into the lymphatic system and adjacent organs limits the choice of effective treatments^4^. The poor prognosis of patients with PC is related to its late detection, high metastatic potential of the cancer, and resistance of the tumor cells to chemotherapy and radiation therapy^5–8^. Therefore, to improve clinical treatment for patients with PC, identifying new biomarkers and potential therapeutic targets is essential.

Reliable chromosome condensation is an essential cellular function that ensures compaction and separation of sister chromosomes, which is important for maintaining genomic integrity during mitosis. Multisubunit protein complexes, known as condensins, play a fundamental role in this process in cooperation with other chromosomal components^9,10^. The two types of condensin complexes present in multicellular organisms^11,12^, condensins I and II, share the same structural maintenance of chromosome (SMC) subunits but have different sets of non-SMC subunits; condensin complex I is comprised of NCAPD2, NCAPG, and NCAPH, whereas condensin complex II is comprised of NCAPG2, NCAPD3, and NCAPH2^9^. Each subunit of condensins I and II is highly conserved in a variety of organisms from yeasts to humans^11^. SMC2 and SMC4 (SMC core subunits) belong to a large family of chromosomal ATPases^9^, and SMC proteins form a heterodimeric structure that adopts a V-shaped structure with two long coiled-coil arms^12^. One of the non-SMC subunits of each of the human condensins, NCAPH in condensin I and NCAPH2 in condensin II, belongs to the kleisin family of proteins^11,13^ and binds the ATP-binding cassette (ABC)-transporter-like ATPase domains at the other end of the coils^14^. A kleisin subunit is composed of conserved N- and C-terminal globular domains separated by a variable linker region in different organisms^13^. The other two non-SMC subunits of each of the human condensins, NCAPD2 and NCAPG in condensin I and NCAP-D3 and NCAPG2 in condensin II, share a structural motif called the HEAT repeat, belonging to HEAT repeat proteins^15,16^. The HEAT repeats are repetitive arrays of short amphiphilic α-helices. According to a recent study, the Ycg1-Brn1 (NCAPG/G2-NCAPH/H2) subcomplex of condensin binds directly to DNA and is essential for the stable association of condensin complexes with chromosomes and consequently condensin function^14^.

The two condensins differ in expression depending on dynamic cell changes during the cell division cycle^17–20^. Condensin II is predominately found in the nucleus during interphase and binds to chromosomes during mitosis^19–21^. Condensin I is located in the cytoplasm and binds to the chromosome after nuclear envelope breakdown^22^. However, recent studies have shown that a small fraction of condensin I remains in the nucleus during interphase and plays a role in gene regulation and chromosome condensation^23^. Condensins I and II differ in their localization within the chromatid axis; condensin II is centrally confined, whereas condensin I localizes to the chromatid diameter from the center, enabling achievement of maximum chromosome compaction upon sister chromatid segregation^23,24^.

Recent studies have also suggested that subunits of condensin complexes I and II can serve as new potential biomarkers and therapeutic targets for some human cancers^25–28^. In particular, the depletion of NCAPH inhibits the proliferation and migration of colon cancer (CC) cells *in vitro* and blocks CC xenograft tumor formation^29^. However, to the best of our knowledge, the effects of NCAPH on PC pathogenesis and its mechanisms have not been previously reported.

Accordingly, in this study, we aimed to evaluate differences in the expression of NCAPH in PC tissues and cell lines compared with that in human pancreatic duct epithelial (HPDE) cells, examined the roles of NCAPH in PC, and assessed the mechanisms through which NCAPH functions in this context.

## Results

### NCAPH expression was upregulated in PC tissues and cell lines

**Figure 1:**
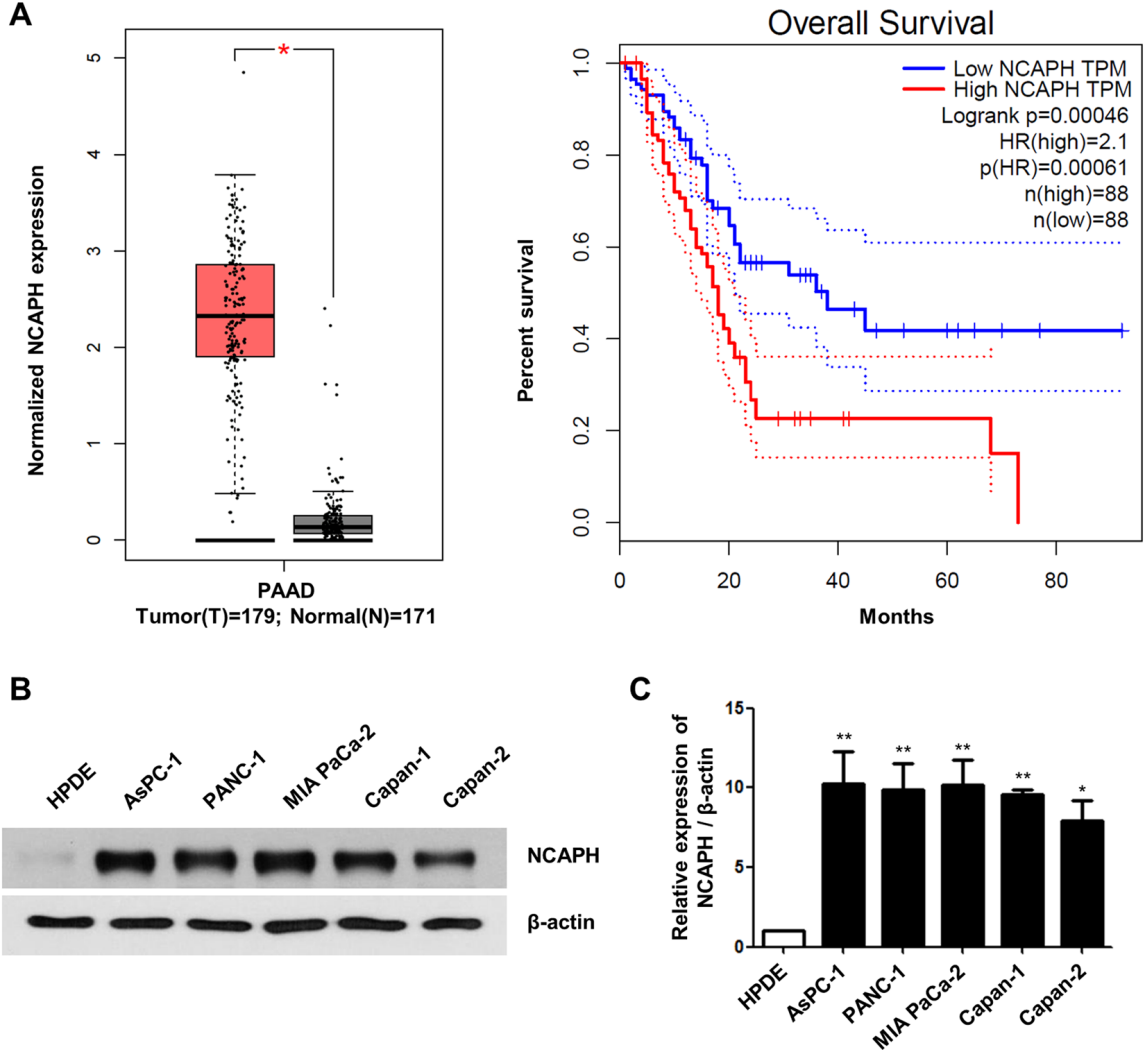
NCAPH expression is elevated in PC tissues and cell lines. (**A)** Expression levels of NCAPH in PC tissues (n = 179) and normal tissues (n = 171) and OS based on the TCGA database. Each dot represents sample expression. The OS of patients with low or high expression levels of NCAPH was analyzed by the Kaplan–Meier method and a log rank test in PAAD. The dotted high line is a sample with a higher expression level than the median value of TPM and is considered a high expression cohort, whereas the dotted low line is a sample with a lower expression level than the median value of TPM and is considered a low expression cohort. Median values are indicated by full lines. *P < 0.005. TCGA, The Cancer Genome Atlas; PAAD, Pancreatic adenocarcinoma; TPM, transcripts per million; OS, overall survival; HR, hazard ratio. (**B)** NCAPH protein expression was analyzed by western blotting in HPDE cells and different PC cell lines (AsPC-1, PANC-1, MIA PaCa-2, Capan-1, and Capan-2 cells). Cell lysates were immunoblotted with the indicated antibodies. (**C)** Frequency of NCAPH protein expression in HPDE and PC cell lines. The NCAPH/β-actin ratio was determined by densitometric analysis using ImageJ. Error bars represent standard deviations of the means of three biological replicates. Values represent means ± SEMs. *P < 0.005, **P < 0.001. Statistical analysis was performed using one-way analysis of variance followed by Bonferroni’s multiple comparison tests.

First, we analyzed the mRNA expression of all subunits of condensin I and II complexes in PC and normal tissues using a publicly available database (The Cancer Genome Atlas [TCGA]). According to the data obtained from TCGA, the expression of NCAPH was significantly higher than that of the condensin subunits in all pancreatic adenocarcinoma (PAAD) tumor types (n = 179) compared with that in their normal tissue counterparts obtained from TCGA and that obtained from GTEx data (n* = *171; Fig. 1A and Supplementary Fig. 1A). In addition, the overall survival (OS) of patients with PC with high NCAPH expression was significantly lower than that of patients with low NCAPH expression (Fig. 1A). Among all subunits of condensins I and II, NCAPH elicited the greatest difference in OS, based on its mRNA expression (Fig. 1A and Supplementary Fig. 1A). We also examined condensin I (NCAPH, NCAPD2, NCAPG) and condensin II (NCAPH2, NCAPG2) protein levels in PC cell lines, including AsPC-1, PANC-1, MIA PaCa-2, Capan-1, and Capan-2 cells. The results showed that protein expression of condensin I rather than II was higher in various PC cell lines compared with its expression in HPDE cells, and NCAPH was markedly upregulated by approximately 10-fold (Fig. 1B,C, and Supplementary Fig. 1B).

### NCAPH knockdown inhibited colony formation and proliferation in PC cells

**Figure 2:**
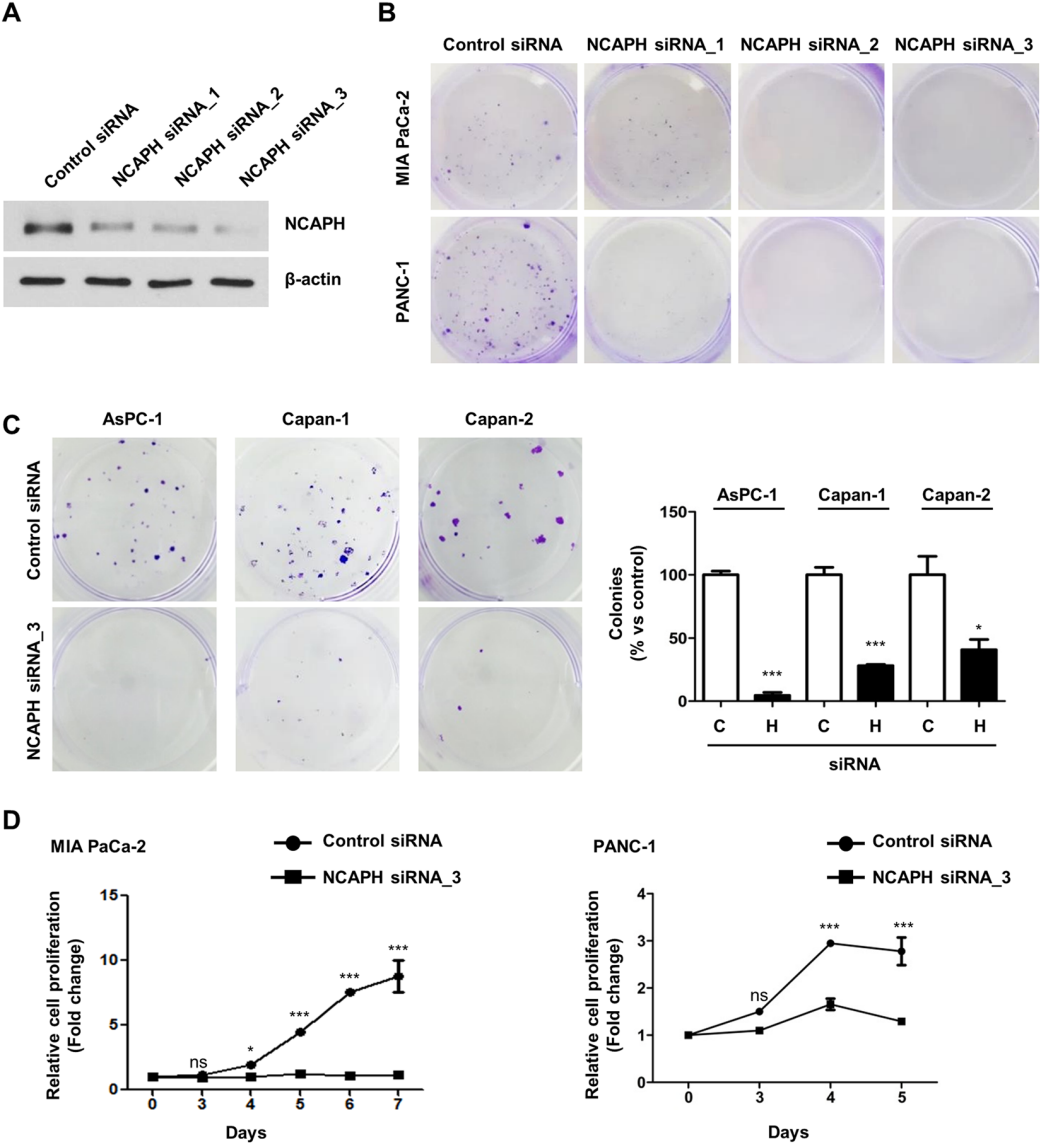
Effects of NCAPH on colony formation ability and proliferation of PC cells. (**A)** MIA PaCa-2 cells were transfected with three different siRNA constructs to knockdown NCAPH expression, and the expression levels of NCAPH protein were measured 48 h later by western blotting. Cell lysates were immunoblotted with the indicated antibodies. (**B)** Colony formation assays were performed on MIA PaCa-2 and PANC-1 cells transfected with the control and three different NCAPH siRNAs (NCAPH siRNA_1, siRNA_2, and siRNA _3). (**C)** Colony formation assays were performed in AsPC-1, Capan-1, and Capan-2 cells with control siRNA and NCAPH siRNA_3. The colonies were imaged and counted. Values represent means ± SEMs. Statistical analysis was performed using unpaired *t*-tests. ***P < 0.0001. (**D)** MTT assays were performed to determine proliferation of control and NCAPH-knockdown PC cells for 5 or 7 days. The curve was based on biological triplicates with values expressed as means ± SEMs. *P < 0.005, ***P < 0.0001. Statistical analysis was performed using two-way analysis of variance with Bonferroni’s multiple comparison tests.

To evaluate the effects of NCAPH knockdown on the survival and growth of PC cells, we employed colony formation assays and 3-(4,5**-**dimethylthiazol**-**2**-**yl)**-**2,5-diphenyltetrazolium bromide (MTT) assays using three NCAPH siRNAs specifically targeting NCAPH. Among these siRNAs, NCAPH siRNA_3 resulted in the most significant inhibition of NCAPH expression and colony formation in MIA PaCa-2 and PANC-1 cells (Fig. 2A,B, and Supplemental Fig. 2A) and was therefore selected for subsequent studies. Indeed, NCAPH knockdown significantly suppressed the colony formation ability of several PC cell lines (e.g., AsPC-1, Capan-1, and Capan-2 cells; Fig. 2C). NCAPH knockdown significantly inhibited the proliferation of PC cell lines, such as MIA PaCa-2 and PANC-1, as demonstrated by MTT assay 5–7 days after siRNA transfection (Fig. 2D). Additionally, we performed a reconstitution experiment after NCAPH siRNA transfection. For this experiment, we constructed a lentiviral vector and NCAPH. When NCAPH was depleted, the proliferation of PC cells was significantly reduced. In contrast, Lenti-NCAPH was able to partially recover the reduced PC cell proliferation (Fig. 2E). Expression of NCAPH protein was also restored by Lenti-NCAPH (Fig. 2F). These results indicated that NCAPH played essential roles in the colony formation and proliferation of PC cells.

### NCAPH knockdown arrested cells in S and G2/M phases and promoted cell apoptosis

**Figure 3:**
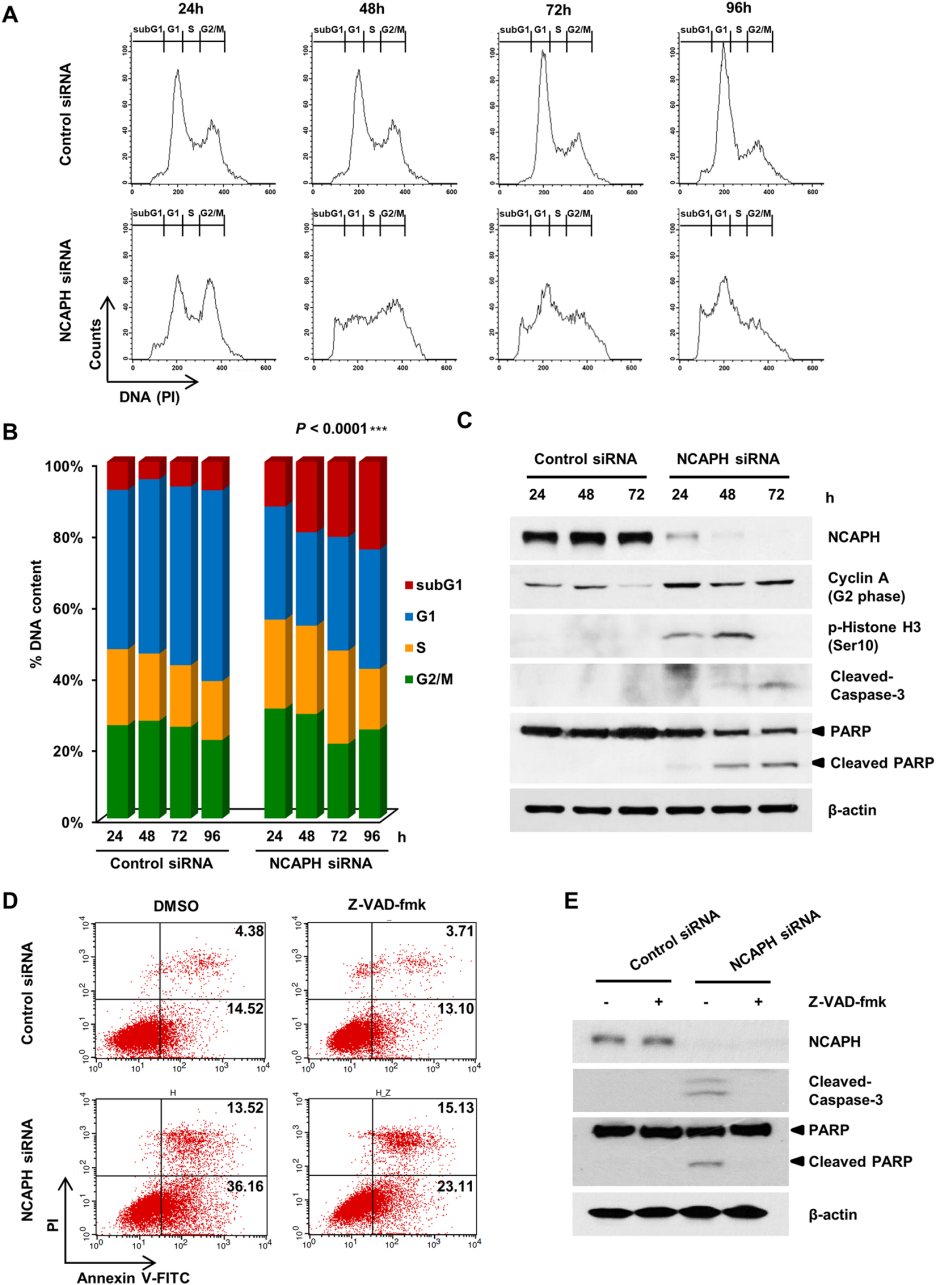
Knockdown of NCAPH induces S and G_2/_M phase arrest and apoptosis. (**A)** MIA PaCa-2 cells were transfected with control siRNA and NCAPH siRNA and then subjected to flow cytometric analysis at 24, 48, 72 and 96 h. The flow cytometry plots and data are representative of at least three separate experiments. (**B)** Bar graph showing the percentages of cells in sub-G_1_ (apoptosis, red), G_1_ (blue), S (orange), and G_2_/M (green) phases. The experiments were repeated three times, and mean values of the results are presented. ***P < 0.0001, two-way analysis of variance. (**C)** Western blot analysis was used to determine the levels of NCAPH, cyclin A, phospho-histone H3, caspase-3, PARP, and β-actin in MIA PaCa-2 cells after transfection with control siRNA or NCAPH siRNA for 24, 48, and 72 h. Cell lysates were immunoblotted with the indicated antibodies. (**D)** Annexin V staining was performed to measure apoptosis using flow cytometry. Z-VAD-Fmk (20 μM) was treated with cells for 24 h. LL (lower left) represents live, LR (lower right) represents early-apoptotic cells, UR (upper right) represents late stage apoptotic cells. (**E)** Western blot analysis was used to determine the levels of NCAPH, caspase-3, PARP, and β-actin in MIA PaCa-2 cells after transfection with control siRNA or NCAPH siRNA. Cells were treated with Z-VAD-Fmk (20 μM) for 24 h.

We performed flow cytometry analysis to investigate the effects of NCAPH on cell cycle regulation and apoptosis in PC cells. Quantification of cells at the subG_1_, G_1_, S, and G_2_/M phases of the cell cycle revealed that the number of NCAPH-knockdown cells accumulated in the S and G_2_/M populations and that the subG_1_ population (apoptotic cells) significantly increased compared with that in the control, whereas the G_1_ cell population was decreased in a concentration-dependent manner (Fig. 3A,B). In addition, we investigated the molecular mechanisms altered by NCAPH knockdown in cell cycle regulation by evaluating the expression of cyclin A and phospho-histone H3 (Ser10) proteins, which regulate the G_2_/M phase transition, by western blotting analysis. Cyclin A and phospho-histone H3 (Ser10) levels significantly increased in NCAPH-knockdown cells 24–48 h after transfection (Fig. 3C). Collectively, these observations suggested that NCAPH knockdown caused S and G_2_/M phase arrest, which could partly explain the reduced viability and enhanced cell death in PC cells.

To further determine whether the cell death observed in NCAPH-knockdown cells was induced by apoptosis, apoptosis levels were investigated using the Annexin V-FITC/PI double staining technique. NCAPH knockdown by siRNA was observed in the early/late apoptosis induction effect (UR and LR quadrants; Fig. 3D). We also identified caspase-3 activity and poly-ADP ribose polymerase (PARP) cleavage events to ensure apoptotic cell death^30^ and found that caspase-3 activation and PARP cleavage were significantly increased in NCAPH-knockdown cells (Fig. 3C). To confirm these results, cells were incubated with 20 μM Z-VAD-Fmk, a pan-caspase inhibitor, for 24 h, which was able to suppress the early apoptosis induced by NCAPH knockdown (LR quadrant; Fig. 3D). Additionally, protein expression of caspase-3 and cleaved PARP was also significantly decreased by Z-VAD-Fmk (Fig. 3E). These data clearly indicated the partial involvement of caspase-dependent apoptosis in NCAPH knockdown-induced cell death.

### NCAPH was involved in progression of mature chromosome condensation (MCC) from poorly condensed chromosomes

**Figure 4:**
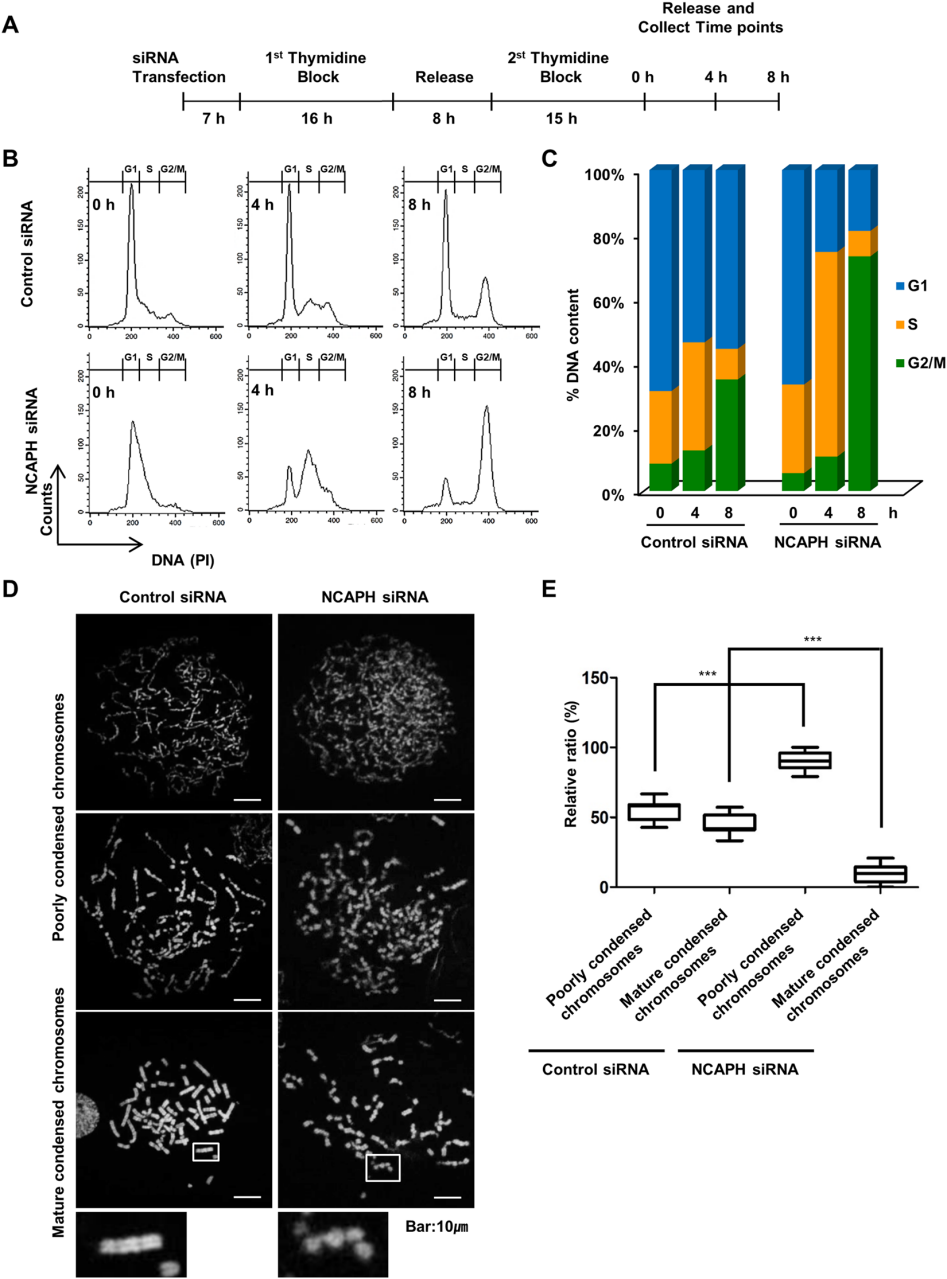
NCAPH-knockdown cells fail to progress of mature chromosome condensation (MCC) from poorly condensed chromosomes. (**A)** General outline of the procedure followed for thymidine double block synchronization of MIA PaCa-2 cells and their cell cycle analysis. Cells were transfected with the indicated siRNAs, and after release from the second thymidine block, cells were assayed at different time points ranging from 0 to 8 h. (**B)** Cells were harvested for PI staining and analyzed by flow cytometry to determine the cell cycle fraction at 0, 4, and 8 h after release. Flow cytometry plots and data are representative of at least three separate experiments. (**C)** The percentages of cells in G_1_, S, and G_2_/M phases are indicated. (**D,E)** Frequencies of poorly condensed chromosomes and mature condensed chromosomes cells in control and NCAPH-knockdown cells. Cells were transfected with the indicated siRNA, treated with one thymidine block (36 h), released for 3 h and then harvested. For accurate quantification, more than 200 cells captured in at least three different fields were analyzed. Values represent means ± SEMs. *P < 0.005, ***P < 0.0001. Statistical analysis was performed using one-way analysis of variance followed by Bonferroni’s multiple comparison tests.

Accumulation of cell ratios at the S and G_2_/M phases indicated that these cells were not able to successfully undergo mitosis when the chromosomes could not be separated before the formation of two daughter cells. To better determine whether the S and G_2_/M phase accumulation previously observed in NCAPH-knockdown cells was a result of a delay or block in cell cycle progression, we investigated cell cycle progression over time after cell release from a double thymidine block (Fig. 4A). Thymidine is a pyrimidine deoxynucleoside that can be used to synchronize cells at the G_1_/S transition. Cell synchronization using double thymidine blocks in control cells allows entry into S phase (0–4 h) and promotes synchronous mitosis (G_2_/M phase: 7–8 h). NCAPH was depleted by siRNA, and then double thymidine block was used to prevent entry of cells into G_1_/S phase. Synchronized cells at different phases were harvested at 0, 4, and 8 h after release from the thymidine block (Fig. 4A). As expected, most cells in the control group had a DNA content consistent with being blocked at either G_1_ or S phase followed by double thymidine treatment (Fig. 4B,C), and the number of cells with a DNA content consistent with S phase increased (22.1% to 32.9%) at 4 h and they progressed to the G_2_/M phase (8.3% to 32.9%) at 8 h after release from the second thymidine block (Fig. 4B,C). In comparison, the cell cycle profile for NCAPH-knockdown cells showed an increase in cells with a DNA content consistent with S phase (26.8% to 62.7%) at 4 h, with a significant proportion of cells maintained at G_2_/M phase (5.4% to 65.8%) at 8 h after release from the second thymidine block (Fig. 4B,C). However, when we performed these experiments on HPDE cells, no differences in cell cycle progression between control siRNA and NCAPH siRNA treatment were observed, as observed in PC cells (Supplemental Fig. 3A). These results indicated that NCAPH may influence the S and G_2_/M phases in PC cells.

Next, to determine when structural chromosome aberrations occurred during cell division, we examined chromosomal breaks, segmentation, and aberrations in control and NCAPH-knockdown cells. In this experiment, thymidine block was performed in control or NCAPH-knockdown MIA PaCa-2 cells, and the chromosomal structure of cells released for 3 h was confirmed by metaphase chromosome spreading assays. Surprisingly, knockdown of NCAPH did not lead to the progression of MCC from poorly condensed chromosomes (Fig. 4D). Furthermore, in NCAPH-knockdown cells, only 9.7% of cells progressed to MCC from poorly condensed chromosomes, as compared with 45.8–54.1% of control cells (Fig. 4E). These results indicated that NCAPH was vital for the progression of MCC from poorly condensed chromosomes.

### NCAPH knockdown promoted chromosomal aberrations and DNA damage

**Figure 5:**
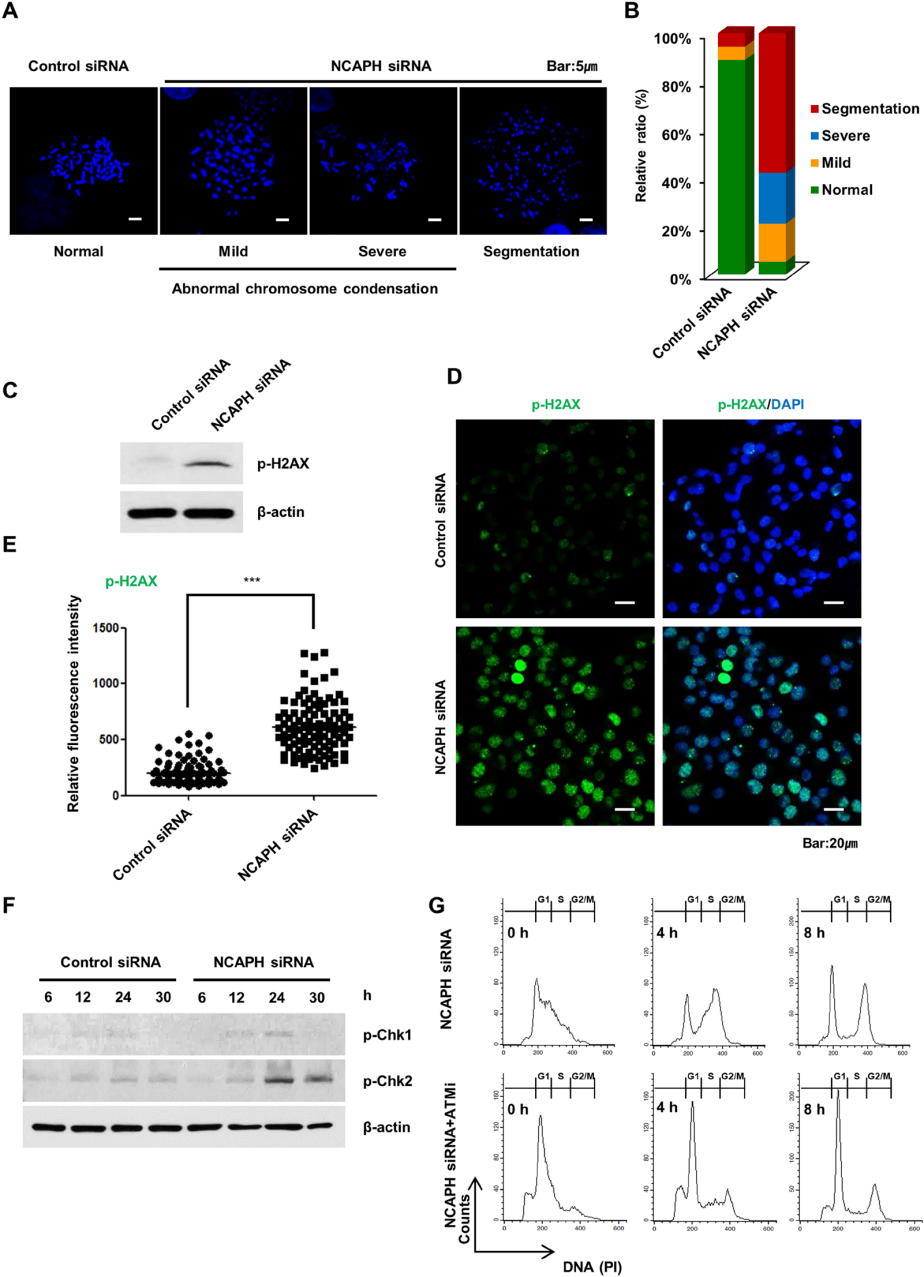
Knockdown of NCAPH induces chromosomal aberrations and DNA damage. (**A,B)** To confirm the chromosome morphology, MIA PaCa-2 cells were transfected with control siRNA or NCAPH siRNA and arrested at metaphase by colcemid treatment for 4 h. The cells were spread onto slides, extracted, fixed, and stained with DAPI (blue). For accurate quantification, more than 50 cells captured in at three different fields were analyzed. Scale bar, 5 µm. (**C)** Western blot analysis of phospho-H2AX expression in control and NCAPH-knockdown cells. Cell lysates were immunoblotted with the indicated antibodies. (**D)** Phospho-H2AX fluorescence pattern (green) in control and NCAPH-knockdown cells was observed by confocal microscopy. DNA was stained using DAPI (blue). Scale bar, 20 µm. (**E)** Frequency of phospho-H2AX fluorescence intensity. For accurate quantification, more than 100 cells captured in at least two different fields were analyzed. Values represent means ± SEMs. ***P < 0.0001, two-way analysis of variance. (**F**) Western blot analysis showing levels of phospho-Chk1 and phospho-Chk2 activation in response to DNA damage in MIA PaCa-2 cells after transfection with control siRNA and NCAPH siRNA for 6, 12, 24, and 30 h. (**G)** Cells were harvested for PI staining and analyzed by flow cytometry to determine the cell cycle fraction after release from the second thymidine block at 0, 4, and 8 h. ATMi (13 μM) was treated with a second thymidine block.

In *Xenopus* egg extracts, as condensins I and II are forced to be smaller, chromosomes become shorter and thicker. Condensin I is involved in lateral compaction, and condensin II is involved in axial shortening^31^. Additionally, in chicken DT40 cells, mitotic chromosomes are wide and short owing to depletion of condensin I, and chromosomes of condensin II-depleted cells appear to be more extended and lack axial stiffness^32^. To elucidate how mitotic chromosome structures are affected by NCAPH knockdown, we performed chromosome spreading assays in MIA PaCa-2 and HeLa cells. Similar to the previous report, shortening and thickening of chromosomes was observed in both types of cells (Supplementary Fig. 4A). However, upon specifically staining with anti-NCAPH antibodies and 4′,6-diamidino-2-phenylindole (DAPI) in MIA-PaCa-2 cells, NCAPH was detectable along the chromatid axis in cells of the control group but not in cells of the NCAPH-knockdown group, and the twisted and segmented chromosome morphology was observed in the NCAPH-knockdown group (Supplementary Fig. 4B). When measuring the number of structural chromosome aberrations in NCAPH-knockdown cells compared with those in control cells, we observed a significant increase (23.7% versus 75.2%, respectively; Supplementary Fig. 4C). To define chromosomal structures more clearly, we divided the state of the chromosomal structures into normal or abnormal chromosome condensations and classified them as mild, severe, or segmentation. The abnormal chromosome condensation (mild and severe) and segmentation type chromosome morphology were increased in NCAPH-knockdown cells (Fig. 5A,B). Additionally, we sought to determine whether the structural chromosome aberrations in NCAPH-knockdown cells were associated with DNA damage responses. To establish the presence of DNA damage, we monitored the appearance of DNA damage foci using antibodies detecting phosphorylated H2AX at S139 (phospho-H2AX), a marker of DNA double-strand breaks (DSBs). Western blot and immunofluorescence analyses showed that the levels of phospho-H2AX were higher in NCAPH-knockdown cells than in control cells (Fig. 5C–E). Moreover, phospho-H2AX was more abundant in NCAPH-knockdown cells than in control cells.

Next, we determined whether G_2_/M phase cell cycle arrest was caused by DNA damage induced by NCAPH knockdown. The results showed that the levels of phospho-Chk1 and phospho-Chk2, which are DNA damage markers, were increased 24 and 30 h after NCAPH knockdown (Fig. 5F). In NCAPH-knockdown cells, the signals of phospho-Chk2 increased significantly compared with the signals of phospho-Chk1 (Fig. 5F). Moreover, to further define the role of ATM kinase in Chk2 phosphorylation in cell cycle defects induced by NCAPH knockdown, cell cycle changes were compared with those in cells when treated with thymidine double blocks alone or with a second thymidine block using an ATM inhibitor (KU-55933, ATMi). The result showed that the G_2_/M phase arrest in NCAPH-knockdown cells was significantly reduced by the ATM inhibitor (Fig. 5G). These results demonstrated that knockdown of NCAPH induced structural chromosome aberrations, which were associated with DNA damage, through the Chk1/Chk2 signaling pathway in PC cells.

## Discussion

In this study, we reached the following conclusions: (1) NCAPH was upregulated in PC tissues and cell lines; (2) knockdown of NCAPH induced cell cycle arrest and cell apoptosis; (3) NCAPH was important for proper progression of MCC from poorly condensed chromosomes; and (4) NCAPH knockdown induced DNA damage though the Chk1/Chk2 signaling pathway. To the best of our knowledge, this is the first report demonstrating that chromosomal aberrations and DNA damage were induced in NCAPH-knockdown PC cells through the Chk1/Chk2 signaling pathway.

Recent studies have evaluated the relationships between condensin complexes and cancers^25–29^. For example, NCAPH knockdown inhibits proliferation and invasion of CC cells^29^. In hepatocellular carcinoma, lack of NCAPG reduces cell viability, increases apoptosis, and arrests cells in the S phase of the cell cycle^26^. In this study, we focused on NCAPH because its expression was increased in PC tissues and cell lines. Although NCAPH has been shown to be associated with cell invasion and proliferation in CC, its functional role in PC had not yet been evaluated. We found that cell cycle arrest and cell apoptosis were increased by NCAPH knockdown, and apoptosis occurred via the intrinsic apoptotic pathway with loss of mitochondrial potential, activation of caspase-3, and generation of the cleaved form of PARP-1, a typical caspase-3 substrate^33^. In addition, lack of NCAPD2, another subunit of condensin I, also inhibited colony formation by PC cells (Supplemental Fig. 2B,C), confirming that the condensin complex played an important role in PC growth.

To ensure proper chromosome condensation and segregation in mitosis, condensin II promotes the separation of replicated chromosomal loci and initiates structural reorganization of replicated chromosomes during S phase^34^. However, our study showed that when NCAPH (condensin I) was absent, cells were arrested in the S and G_2_/M phase of the cell cycle. Importantly, normal maintenance of the S phase in cell cycle progression could affect the structure of mitotic chromosomes. For example, when mammalian cells in the G_2_ phase are fused with G_1_-phase cells, all cells enter mitosis synchronously. However, the DNA from G_1_-phase cells passes through S phase faster than expected, resulting in less condensed mitotic chromosomes^35^. Importantly, when the G_1_-phase nucleus is fused with the mitotic nucleus, the detected chromosomes showed insufficiently condensed and much longer than normal chromatids^35,36^. These experiments have demonstrated that activities in mitotic cells result in condensation of interphase chromatin but that the structure of the condensed chromosomes is deficient. Moreover, lack of B-Myb, an Myb family transcription factor that plays an essential role during S phase, decreases S-phase progression and increases chromosomal fragmentation and other aberrations^37^. In any case, the completion of DNA replication is associated with proper chromosome condensation. Based on our observations after the knockdown of NCAPH, we hypothesize that NCAPH may have a major role in facilitating correct cell cycle progression through the S and G_2_/M phases and accompanying chromosome condensation. NCAPH knockdown resulted in chromosomal fragmentation and DNA damage responses, which affected the progression of MCC from poorly condensed chromosomes. We demonstrated that NCAPH-knockdown cells were arrested at S and G_2_/M phases of the cell cycle and blocked the progression of MCC from poorly condensed chromosomes, preventing the completion of proper chromosome condensation of PC cells. These findings suggested that NCAPH played an important role in the progression of MCC from poorly condensed chromosomes during the cell cycle process, revealing a novel mechanism through which progression of MCC from poorly condensed chromosomes is regulated by NCAPH.

When DNA damage occurs, cells spend more time undergoing DNA repair processes or apoptosis, particularly if the damage is severe. Therefore, cells have maintained genome integrity by developing complex machinery that includes DNA-damage responses, which detect and repair DNA damage. Generally, there are two types of DNA damage, DSBs and single-strand breaks (SSBs). DSBs are rapidly detected by the Mre11/Rad50/NBS1 complex, which subsequently promotes the activation of ataxia telangiectasia mutated kinase by autophosphorylation^38^. SSBs are sensed by the Rad9/Hus1/Rad1 complex, which activates ataxia telangiectasia and Rad3-related kinase^39^. Following this sensing step, Chk1 and Chk2 amplify the signals from the sensors, phosphorylating a variety of effectors^40^. Additionally, Chk1 monitors S phase replication and mitosis entry^41,42^, and Chk2 triggers the G_1_/S checkpoint and apoptosis^43^. In particular, SMC2 recruitment to mitotic chromosomes is controlled by DNA damage response checkpoints, such as Chk2, which facilitates effective repair of genomic damage^44^. Based on these findings, our data clearly indicated that NCAPH plays a critical role in the response to DNA damage through the increased phosphorylation of Chk1/Chk2 kinases. This finding was confirmed by the results of ATM treatment, which reduced the arrest of cells in G_2_/M phase caused by NCAPH knockdown because ATM is responsible for the phosphorylation of Chk2.

In conclusion, the results of this study confirmed that NCAPH knockdown induced chromosomal aberrations and DNA damage through the Chk1/Chk2 signaling pathway, ultimately resulting in selective cell death through partially caspase-dependent apoptotic signaling in PC cells. Based on our findings, we hypothesize that NCAPH may be associated with human carcinogenesis and could be a potential therapeutic target for human cancer. Further studies are needed to test this hypothesis.

## Methods

### Database analysis

Expression levels of the subunits of condensin I complex in PC and normal pancreas were achieved from Gene Expression Profiling Interactive Analysis (GEPIA; http://gepia.cancer-pku.cn/)^45^, which analyzed 179 PAAD tumors and 171 normal samples from TCGA tumor samples with paired adjacent TCGA normal samples and GTEx normal samples. Transcript per million (TPM) values were calculated, and the expression levels of genes were presented using the Log_2_ (TPM + 1) scale. The cutoff values were 1 for |Log_2_FC| and 0.01 for the *P* value. The OS of patients with PC was also analyzed.

### Cell culture and siRNA knockdown

MIA PaCa-2 (American Type Culture Collection [ATCC] CRL-1420; ATCC, Manassas, VA, USA) and PANC-1 (ATCC CRL-1469) human PDAC cell lines were grown in high-glucose Dulbecco’s modified Eagle’s medium (DMEM). Human PDAC cell lines (AsPC-1, Capan-1, and Capan-2) were grown in RPMI medium. Noncancerous immortalized HPDE cells were obtained from Joo Kyung Park, MD (Samsung Medical Center, Seoul, South Korea). HPDE cells were grown in Defined K-SFM medium. All cell culture media contained 10% fetal bovine serum, 100 U/mL penicillin, and 100 μg/mL streptomycin (Gibco, Life Technologies, Grand Island, NY, USA). To knockdown NCAPH expression, the cells were transfected with siRNA using the Lipofectamine RNAiMAX transfection reagent (Invitrogen, Carlsbad, CA, USA) according to the manufacturer’s instructions. Three different NCAPH siRNAs were prepared^46^. The three NCAPH siRNAs and control siRNA were purchased from Cosmo Bio Co., Ltd. (Tokyo, Japan)

### Cell proliferation and viability assay

Cell proliferation and viability assays were performed using MTT solution. MIA PaCa-2 and PANC-1 cells were seeded into 12-well plates at a density of 1 × 10^4^ cells/well or 8 × 10^4^ cells/well. After adhesion for 24 h, the cells were transfected with NCAPH siRNA or control siRNA and incubated for 5 or 7 days. For colony formation assays, cells were seeded into 6-well plates at a density of 500–1000 cells/well and incubated for 14 days. The colonies were then fixed in 100% methanol, stained with 10% crystal violet, and counted. Each assay was performed in triplicate.

### Chromosome spreading assay

Cells were treated with colcemid (0.1 µg/mL) for 4 h and then harvested. After treatment with 0.075 M KCl, cells were fixed with a drop of freshly made fixative (methanol/acetic acid [3:1]) and placed on glass slides. The slides were dried at room temperature, stained with DAPI (100 ng/mL), and visualized under a confocal microscope (Zeiss LSM710, software ZEN; Zeiss, Germany).

### Immunofluorescence

Cells were grown on Thermo Scientific Nunc Lab-Tek II Chamber Slides, permeabilized with 0.5% triton X-100 for 1 min, and fixed with 4% paraformaldehyde for 10 min. The fixed cells were incubated for 1 h at room temperature with blocking solution (1% bovine serum albumin) and then incubated overnight at 4 °C with primary antibodies. Cells were then incubated with secondary antibodies plus 100 ng/mL DAPI for 3 h. Samples were mounted in Prolong Gold Antifade reagent (Invitrogen), and the results were viewed under a confocal microscope (Zeiss LSM710, software ZEN). The primary antibodies were purchased from commercial sources, as follows: anti-NCAPH (Novus Biological, Littleton, CO, USA), and anti-phosphorylated H2AX at S139 (phospho-H2AX; Millipore, Temecula, CA, USA).

### Western blotting and antibodies

Whole cells were harvested, and proteins were extracted using 1 × RIPA buffer. Protein concentrations were determined using a BCA Protein Assay Kit (Pierce, Rockford, IL, USA). Protein extracts were resuspended in 5 × sample buffer (50 mM tris [pH 6.8], 100 mM dithiothreitol, 2% sodium dodecyl sulfate [SDS], 0.1% bromophenol blue, and 10% glycerol), boiled for 5 min, and subjected to SDS-polyacrylamide gel electrophoresis on 8–15% gels. Separated proteins were transferred to Trans-blot nitrocellulose membranes (Schleicher & Schuell, Keene, NH, USA), and membranes were blocked in 5% skim milk in TBST (10 mM Tris [pH 8.0], 150 mM NaCl, 0.05% tween 20) and incubated with several primary antibodies. The primary antibodies were purchased from commercial sources, as follows: anti-NCAPH (Novus Biological), anti-cyclin A (Cell Signaling Technology, Danvers, MA, USA), anti-phospho-histone H3 pS10 (Abcam, Cambridge, MA, USA), anti-phospho-Chk1 at Ser345 (Cell Signaling Technology), anti-phospho-Chk2 at Thr68 (Cell Signaling Technology), anti-caspase-3 (Cell Signaling Technology), anti-poly (ADP ribose) polymerase (PARP; Cell Signaling Technology), anti-phospho-H2AX (Millipore, Temecula, CA, USA) and anti-β-actin (Cell Signaling Technology). Protein expression was detected based on chemiluminescent signals activated by SuperSignal West Pico Chemiluminescent Substrate (Pierce, Thermo Scientific, USA) after reaction with horse radish peroxidase-tagged secondary antibodies (Jackson Immunoresearch Laboratories, West Grove, PA, USA).

### Fluorescence-assisted cell sorting (FACS) analysis

For cell cycle and DNA content analysis, cultured cells were incubated in trypsin-ethylenediaminetetraacetic acid at 37 °C in an atmosphere containing 5% CO_2_, collected by centrifugation, and washed once with 1 × phosphate-buffered saline. Cells were centrifuged, supernatants were removed, and cells were stained with 50 μg/mL propidium iodide (PI; Sigma-Aldrich, St. Louis, MO, USA) plus 100 U ribonuclease A from bovine pancreas (Sigma-Aldrich). Analysis was performed with a FACSCalibur (BD Biosciences, San Diego, CA, USA) according to standard protocols.

Apoptosis was analyzed by FACS using FITC-conjugated annexin V (BD Biosciences, San Diego, CA, USA) and propidium iodide (PI; Sigma-Aldrich) staining. Analysis was performed with a FACSCalibur (BD Biosciences) according to standard protocols. Inhibition of apoptosis was performed with the pan-caspase inhibitor Z-VAD-FMK (20 µM; R&D Systems, Minneapolis, MN, USA).

### Statistical analysis

All data represent average values obtained in form three independent experiments. Results were statistically analyzed using GraphPad Prism (version 5.0, GraphPad Software Inc., San Diego, CA, USA) and presented as means ± standard errors of the means (SEMs). Statistical analysis was performed using one-way or two-way analysis of variance followed by Bonferroni’s multiple comparison tests.

## Supplementary information

supplementary dataset 1
