# Supplementary material for: Non-SMC condensin I complex subunit H mediates mature chromosome condensation and DNA damage in pancreatic cancer cells
Source: Sci Rep. 2019 Nov 29;9:17889. doi: 10.1038/s41598-019-54478-3 (PMC6884527; doi:10.1038/s41598-019-54478-3)
Supplement: Supplementary file 1 — supplementary dataset 1 [file 41598_2019_54478_MOESM1_ESM.pdf]

Non-SMC condensin I complex subunit H mediates mature chromosome condensation and DNA damage in pancreatic cancer cells

Jae Hyoeng Kim[1*], Yuna Youn[1], Kyung-Tae Kim[2], Jang Gyubeom[2], Jin-Hyeok Hwang[1,3*]

[1] Department of Internal Medicine, Seoul National University Bundang Hospital, Seongnam-si, Gyeonggi-do, 13620, Republic of Korea

[2] Research Institute, National Cancer Center, Goyang-si, Gyeonggi-do, 10408, Republic of Korea

[3] Department of Internal Medicine, Seoul National University College of Medicine, Seoul, 03080, Republic of Korea

A

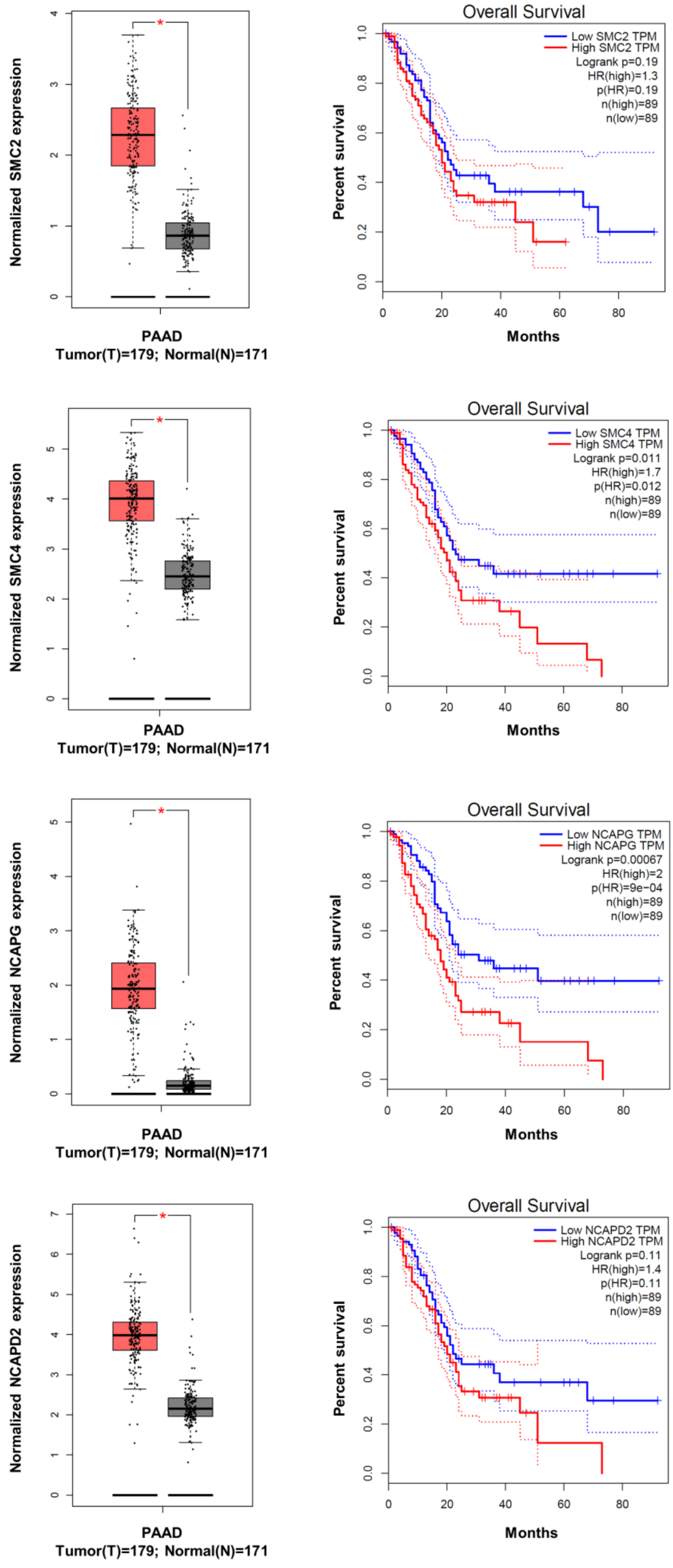

Normalized SMC2 expression
PAAD
Tumor(T)=179; Normal(N)=171
Overall Survival
Low SMC2 TPM
High SMC2 TPM
Logrank p=0.19
HR(high)=1.3
p(HR)=0.19
n(high)=89
n(low)=89
Percent survival
Months
Normalized SMC4 expression
PAAD
Tumor(T)=179; Normal(N)=171
Overall Survival
Low SMC4 TPM
High SMC4 TPM
Logrank p=0.011
HR(high)=1.7
p(HR)=0.012
n(high)=89
n(low)=89
Percent survival
Months
Normalized NCAPG expression
PAAD
Tumor(T)=179; Normal(N)=171
Overall Survival
Low NCAPG TPM
High NCAPG TPM
Logrank p=0.00067
HR(high)=2
p(HR)=9e−04
n(high)=89
n(low)=89
Percent survival
Months
Normalized NCAPD2 expression
PAAD
Tumor(T)=179; Normal(N)=171
Overall Survival
Low NCAPD2 TPM
High NCAPD2 TPM
Logrank p=0.11
HR(high)=1.4
p(HR)=0.11
n(high)=89
n(low)=89
Percent survival
Months

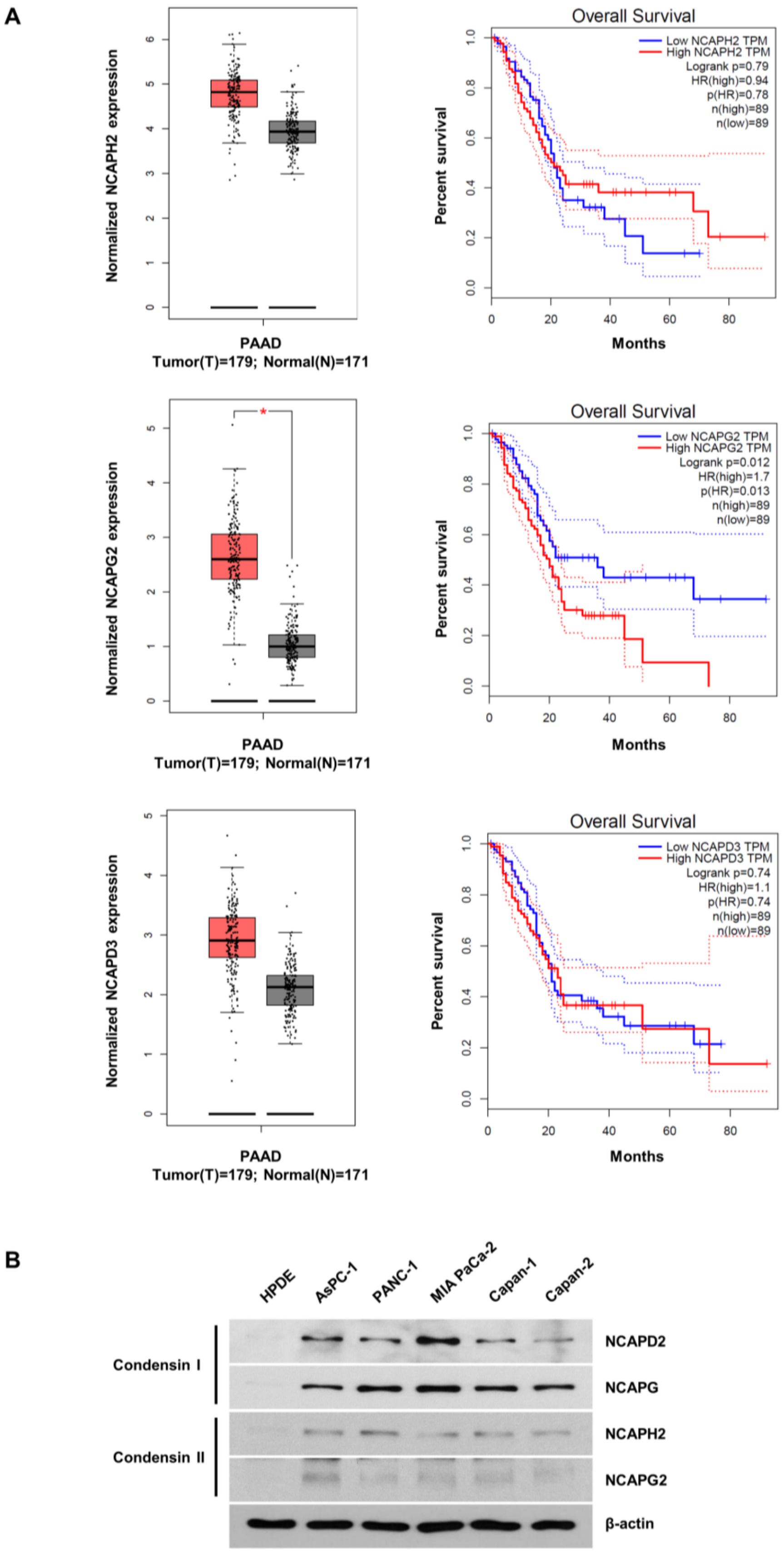
A
Normalized NCAPH2 expression
PAAD
Tumor(T)=179; Normal(N)=171
Overall Survival
Low NCAPH2 TPM
High NCAPH2 TPM
Logrank p=0.79
HR(high)=0.94
p(HR)=0.78
n(high)=89
n(low)=89
Percent survival
Months
Normalized NCAPG2 expression
PAAD
Tumor(T)=179; Normal(N)=171
Overall Survival
Low NCAPG2 TPM
High NCAPG2 TPM
Logrank p=0.012
HR(high)=1.7
p(HR)=0.013
n(high)=89
n(low)=89
Percent survival
Months
Normalized NCAPD3 expression
PAAD
Tumor(T)=179; Normal(N)=171
Overall Survival
Low NCAPD3 TPM
High NCAPD3 TPM
Logrank p=0.74
HR(high)=1.1
p(HR)=0.74
n(high)=89
n(low)=89
Percent survival
Months
B
HPDE
AsPC-1
PANC-1
MIA PaCa-2
Capan-1
Capan-2
Condensin I
Condensin II
NCAPD2
NCAPG
NCAPH2
NCAPG2
β-actin

Supplementary Figure 1. Expression of condensin I and II subunits in PC tissues and overall survival based on GEPIA data. A, Expression levels of subunits of condensin I and II subunits in PC tissues (*n* = 179) and normal tissues (*n* = 171) and overall survival (OS) based on the TCGA database were analyzed. Each dot represents sample expression. OS of patients with low or high expression levels of subunits of condensin I and II complexes was analyzed by the Kaplan–Meier method and log rank tests (PAAD: pancreas adenocarcinoma). $^*P < 0.005$. **B,** NCAPD2, NCAPG, NCAPH2 and NCAPG2 protein expressions were analyzed by western blotting in human pancreatic duct epithelial (HPDE) cells and different PC cell lines (AsPC-1, PANC-1, MIA PaCa-2, Capan-1, and Capan-2 cells). Cell lysates were immunoblotted with the indicated antibodies.

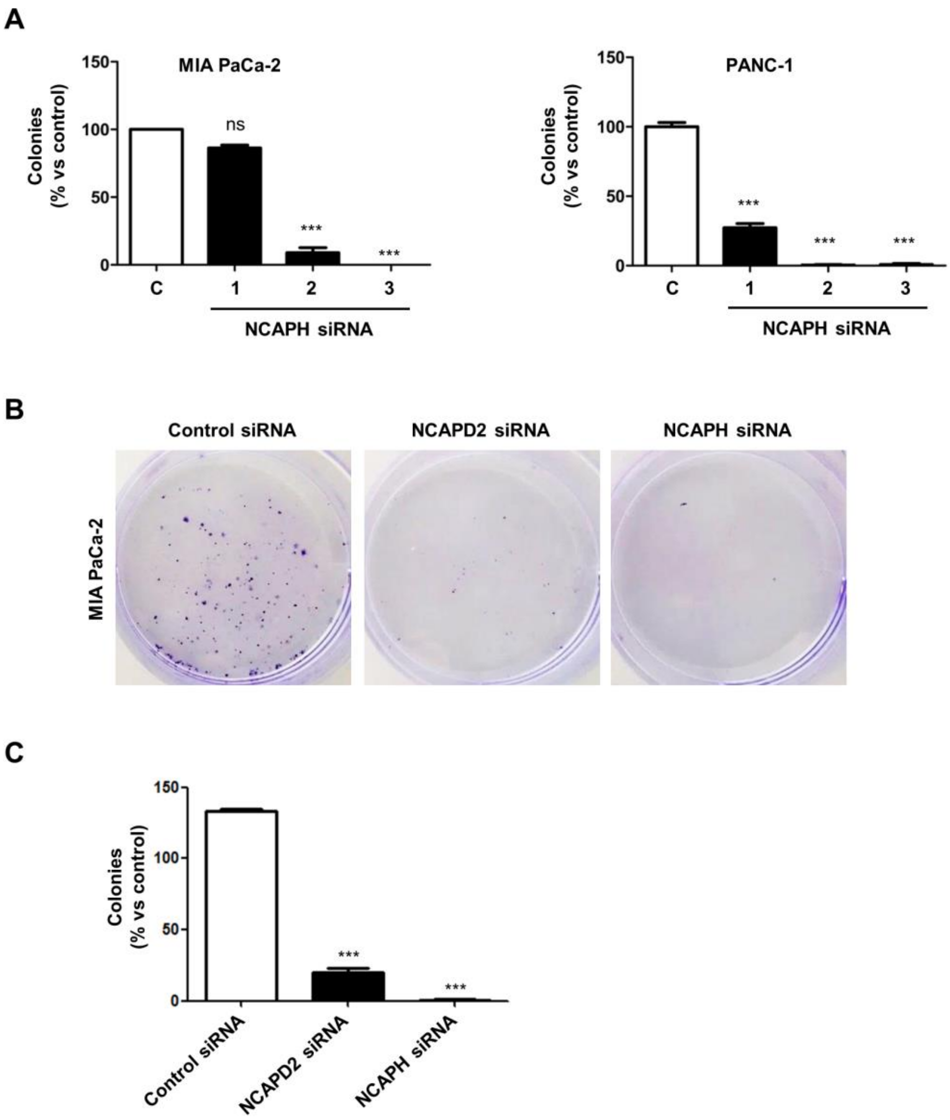

Supplementary Figure 2. NCAPH- or NCAPD2-knockdown PC cells inhibit colony formation. A, The colonies of Figure 2B were counted. **B and C,** The colonies were imaged and counted. Values represent means ± SEMs. ***P < 0.0001. Statistical analysis was performed using one-way analysis of variance followed by Bonferroni's multiple comparison tests.

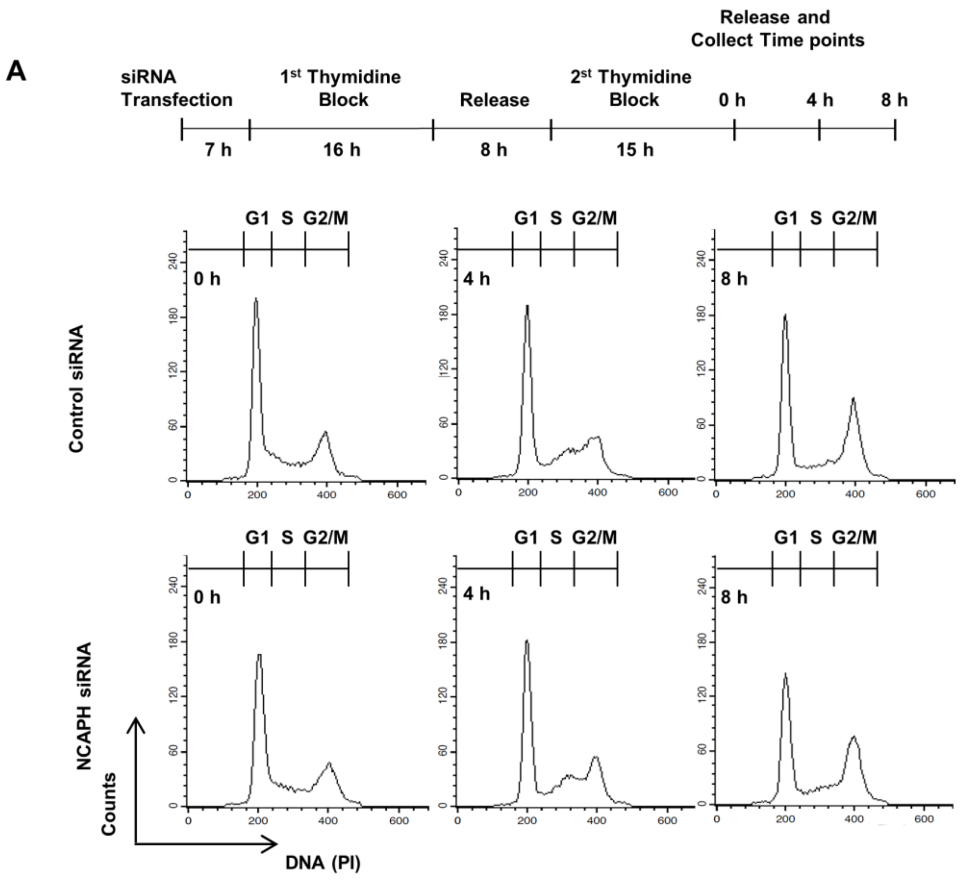

Supplementary Figure 3. Knockdown of NCAPH in HPDE cells does not affect cell cycle processes. A, HPDE cells were transfected with the indicated siRNAs, and after release from the second thymidine block, cells were assayed at different time points ranging from 0 to 8 h. The HPDE cells were harvested for propidium iodide (PI) staining and analyzed by flow cytometry to determine the cell cycle fraction at 0, 4, and 8 h after release.

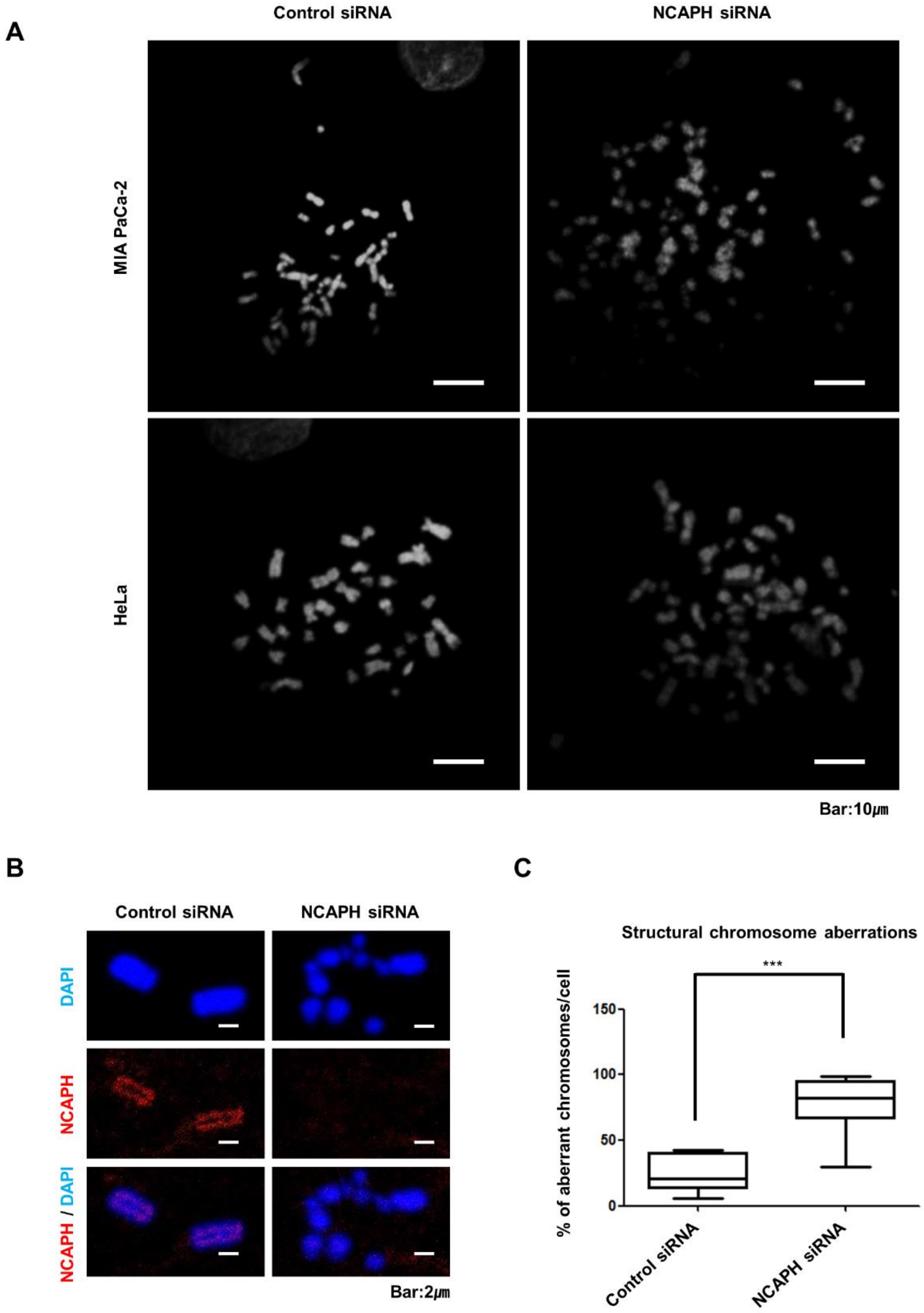

Supplementary Figure 4. Confirmation of chromosome morphology by NCAPH knockdown in MIA PaCa-2 and HeLa cells. A, MIA PaCa-2 and HeLa cells were transfected with control siRNA or NCAPH siRNA and arrested at metaphase by colcemid

treatment for 4 h. DNA was stained using DAPI (grey). Individual chromosomes: Scale bar, 10 μm. **B,** The control- or NCAPH-knockdown cells (MIA PaCa-2) were spread onto slides, extracted, fixed, and stained with anti-NCAPH antibodies (red). DNA was stained using DAPI (blue). Individual chromosomes: Scale bar, 2 μm. **C,** Frequency of abnormal chromosome morphology in control- and NCAPH-knockdown cells. For accurate quantification, more than 800 chromosomes (more than 10 cells) captured in at least three different fields were analyzed. Values represent means ± SEMs. ***P < 0.0001, two-way analysis of variance.

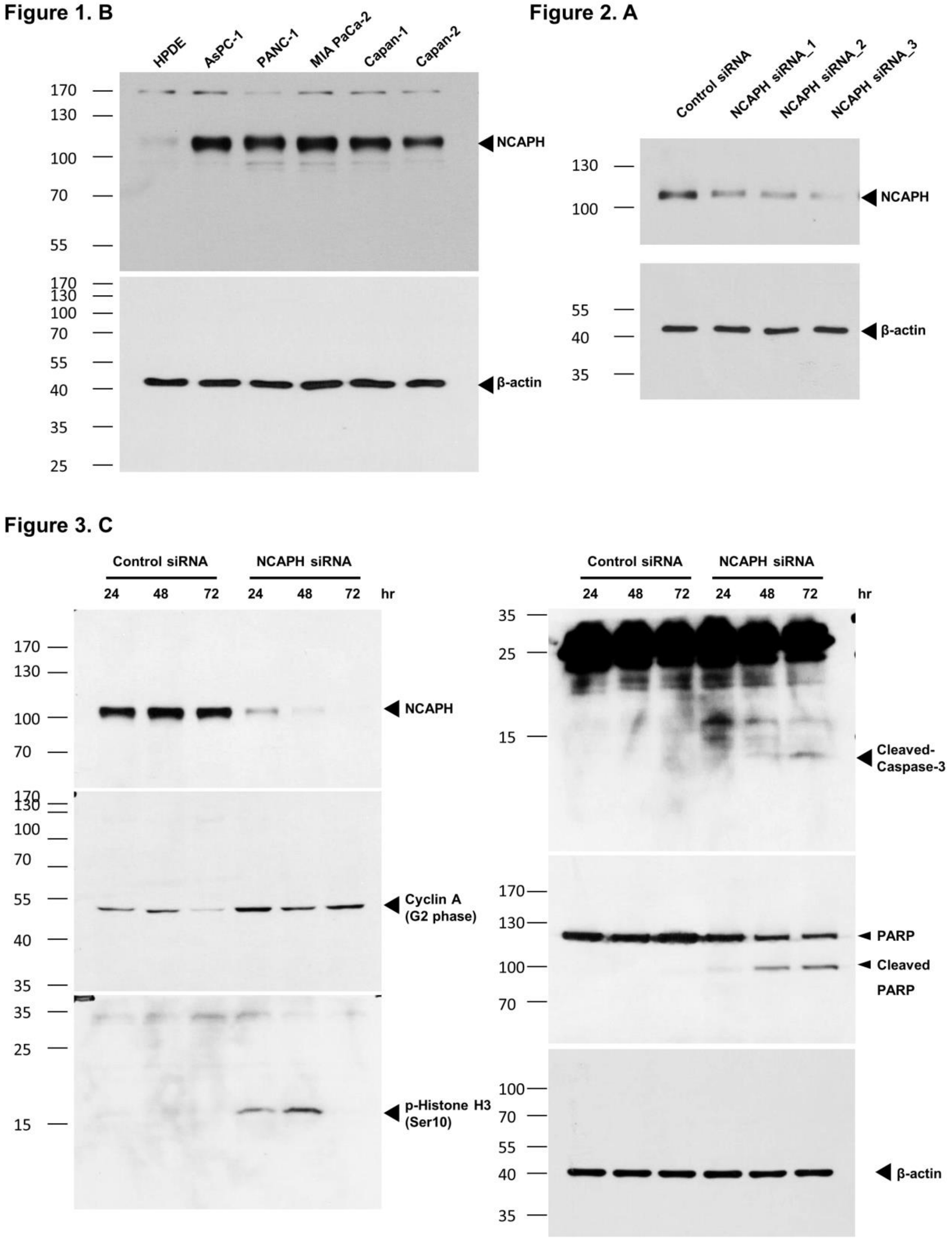

Supplementary Figure 5. Full-length immunoblots.

Figure 3. E

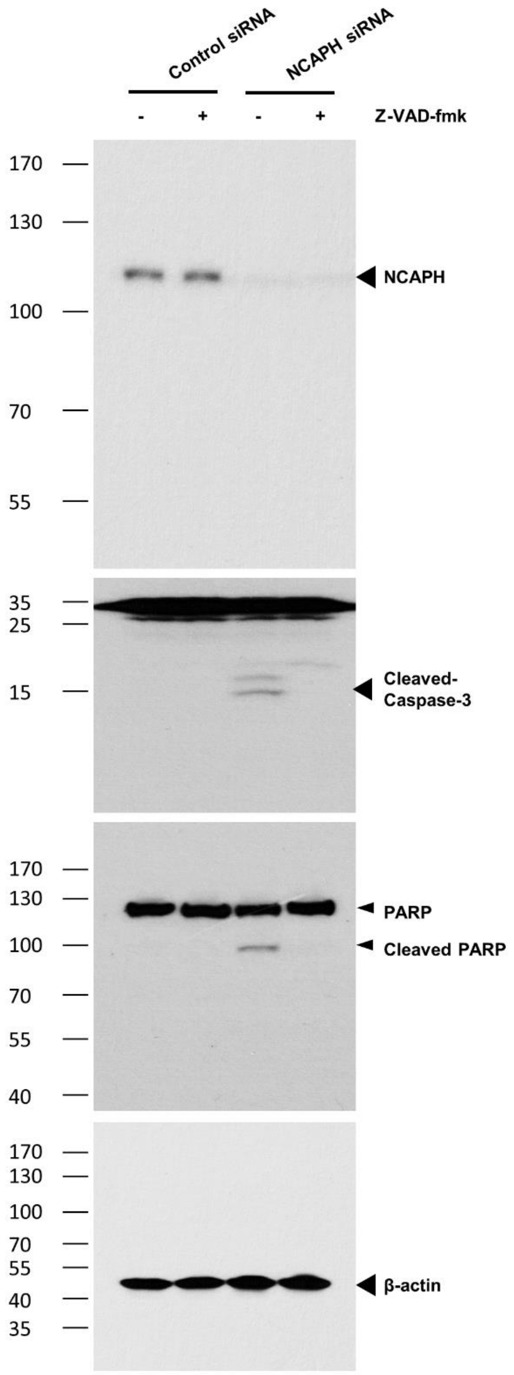

Figure 5. C

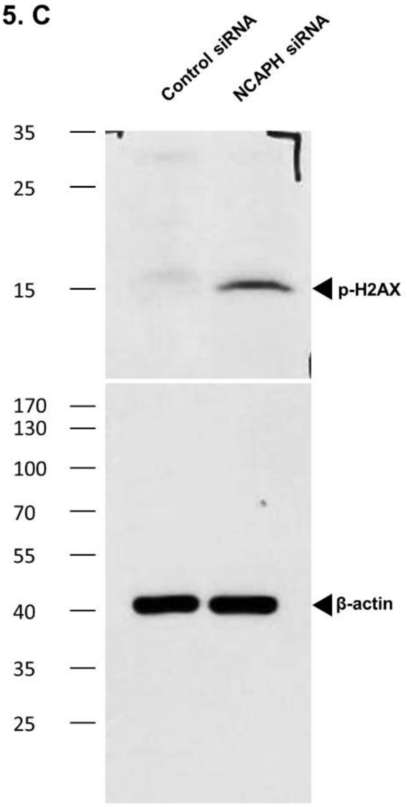

Supplementary Figure 5. Continued

Figure 5. F

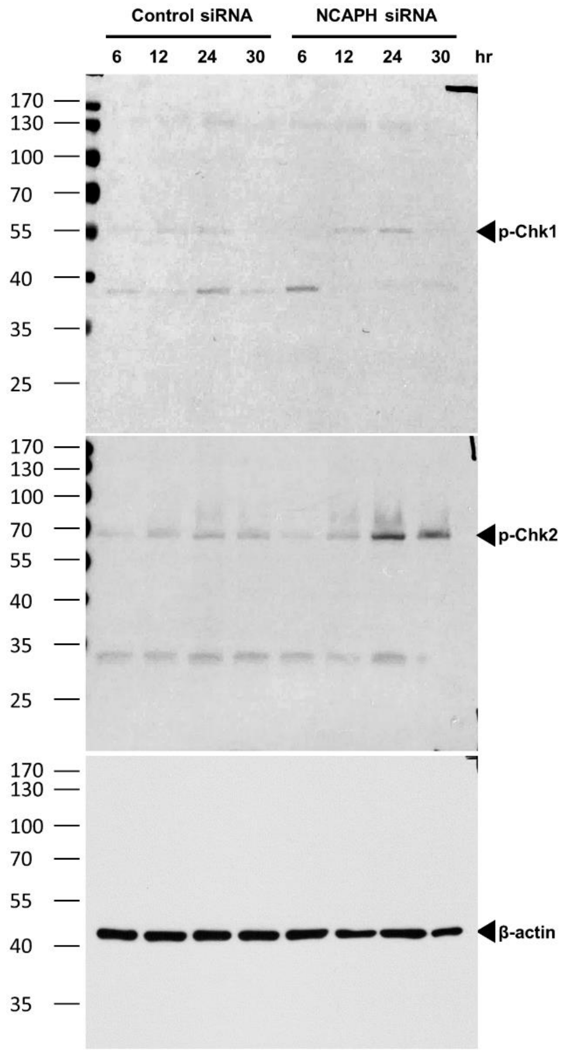

Supplementary Figure 5. Continued

Supplementary Figure 1. B

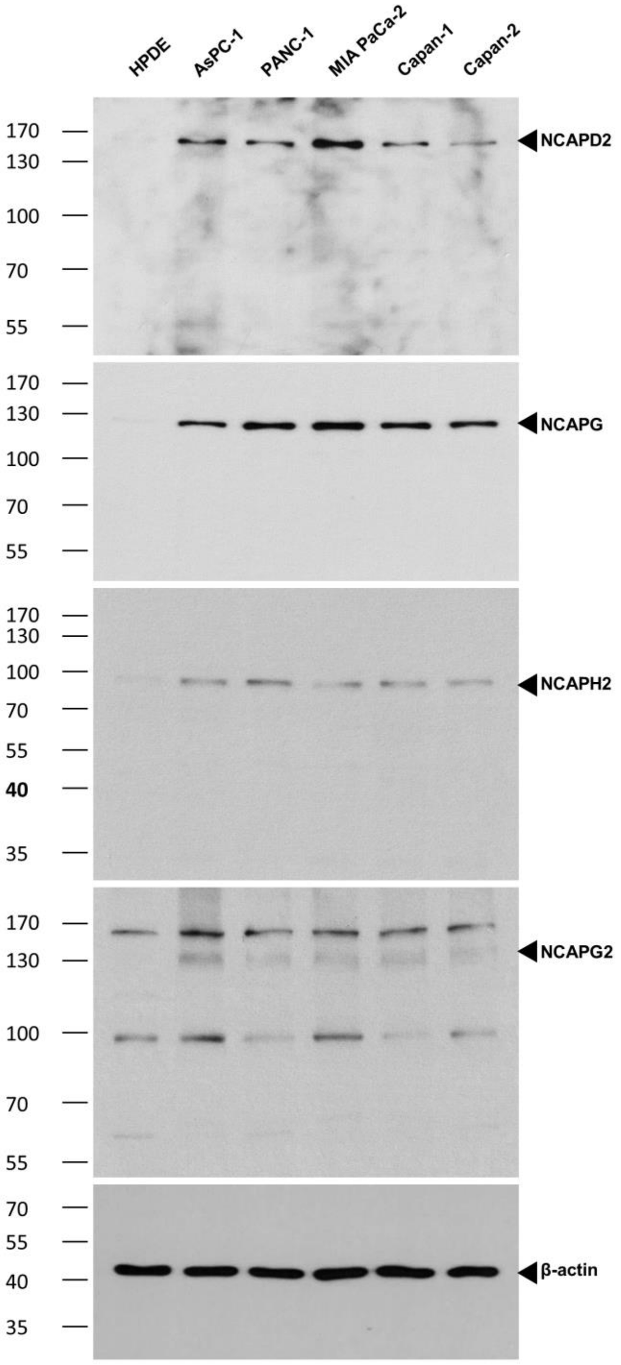

Supplementary Figure 5. Continued
